# Dosimetric impact of target definitions on normal structures in head and neck cancer

**DOI:** 10.1186/1758-3284-3-34

**Published:** 2011-08-11

**Authors:** Micah T Monaghan, James A Bonner, Philip E Schaner, Jimmy J Caudell

**Affiliations:** 1 Department of Radiation Oncology, The University of Alabama at Birmingham, 1700 Sixth Avenue South, Birmingham, AL 35249, USA; 2 Radiation Oncology of North Mississippi, 620 Crossover Road, Tupelo, MS 38801, USA; 3 Section of Radiation Oncology, Dartmouth Hitchcock Medical Center, 1 Medical Center Drive, Lebanon, NH 03756, USA; 4 Department of Radiation Oncology, University of Mississippi Medical Center, 350 W. Woodrow Wilson Drive, Suite 1600, Jackson, MS 39213, USA

**Keywords:** Head and neck cancer, intensity modulated radiation therapy, normal tissue sparing

## Abstract

**Background and Purpose:**

Previous work by our group suggests smaller target volumes may result in equivalent locoregional control for head and neck cancer. We evaluated whether smaller target volumes may also result in improved normal tissue sparing.

**Methods and Materials:**

Ten patients with Stage III-IV head and neck cancer were contoured and planned according to target definitions in RTOG 0522 in a two dose level plan (RTOG), as well as a three dose level plan, using smaller target volumes and an intermediate dose prescription (3Dose). Plans were compared for coverage of targets and sparing of normal tissues

**Results:**

The high dose target, elective nodal target, and total volume targeted were significantly smaller in 3Dose plans (p < 0.001). There was no difference in volume receiving 100% of each prescription level in RTOG or 3Dose plans. Mean dose to contralateral parotid, mandible, larynx, and inferior pharyngeal constrictor, and maximum dose to brainstem were significantly lower in 3Dose plans. There was no significant difference in maximum dose to spinal cord or volume of tissue not otherwise specified receiving 70 Gy.

**Conclusions:**

Smaller target volumes with the addition of an intermediate dose volume results in improved sparing of most normal tissues.

## Introduction

As treatments for locally advanced head and neck cancer (LAHNC) have become more aggressive through the use of altered fractionation radiotherapy and concomitant chemotherapy, concern has grown regarding the risk of late toxicities in normal tissue.

The use of intensity-modulated radiation therapy (IMRT) makes it possible to more effectively spare normal tissues, such as the parotid gland. Contouring and construction of targets is extremely important in planning IMRT. Currently, no consensus exists regarding the appropriate extent of margins for the clinical target volume (CTV).

In a recent paper, we showed that there was no significant difference in locoregional control between anatomic and volumetric expansions of gross tumor volumes (GTV) [1]. Additionally, smaller volumetric expansions appeared to result in similar locoregional control as larger volumetric expansions. A number of the patients reported in that study had a direct GTV to planning target volume (PTV) expansion of 4-6 mm without the use of a CTV-high dose; many of these employed the use of a CTV-intermediate dose (CTV-ID) to account for possible microscopic extension. No significant difference in locoregional control was found for PTV-high dose (PTV-HD) expansions of 4-6 mm, 10-15 mm, or >15 mm.

Theoretically, the definition of radiation targets should impact the ability of a radiation oncologist to spare normal tissues. The recently completed Radiation Therapy and Oncology Group trial 0522 (RTOG 0522) for LAHNC employed target volumes intended to provide a regimen that was based on historical knowledge and could be implemented in a large cooperative group trial. The target volumes stipulated by the RTOG 0522 protocol tend to render volumes that would be at the upper limits of the volumes that were studied in the above mentioned investigation of Caudell et al. In the current report, we compare the RTOG 0522 target definitions with smaller target volumes and their outcome on normal tissue sparing.

## Materials and methods

**Figure 1:**
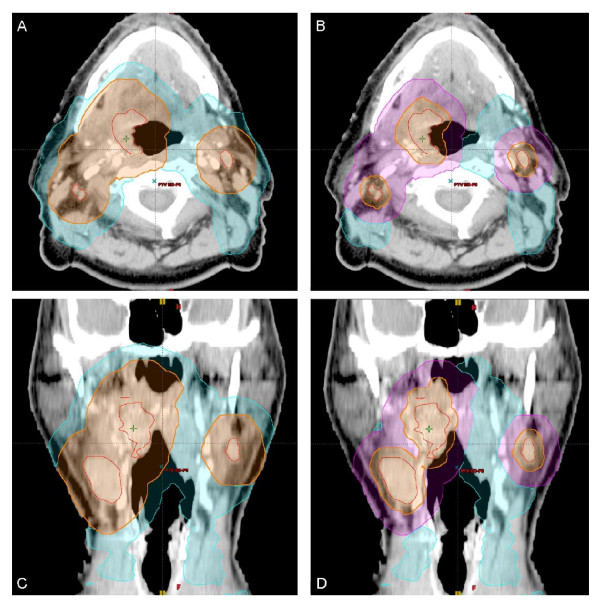
**Representative patient demonstrating variation in planning target volumes (PTV) for two dose (RTOG) and three dose (3Dose) plans**. Axial (A-B) and coronal (C-D) computed tomography images shown. The gross tumor volume (GTV, red line) is identical for each plan. The PTV-high dose (PTV-HD, orange) and PTV-elective dose (PTV-ED, cyan) are presented for RTOG in A and C, respectively. These volumes plus PTV-intermediate dose (PTV-ID, magenta) are displayed for 3Dose in B and D.

Ten patients with LAHNC of the oropharynx, hypopharyx, or larynx (Table 1) were identified who were treated on RTOG 0522 (RTOG). RTOG contour definitions are duplicated in Table 2. The same 10 patients were also contoured and planned using smaller target volumes and an intermediate dose level (3Dose, Table 2). The major difference in the 3Dose volumes was the exclusion of a CTV for the high dose volume, with the intermediate volume being identical to the high dose target in the RTOG plan. The normal tissue contours, GTV, and elective nodal volumes were identical between patients' plans. Identical PTV volumetric expansions were utilized for each patient's plan, though this did vary between patients (3 - 5 mm). Each PTV was modified to exclude 1 - 5 mm of skin, and this was identical for each patient's plans. Figure 1 is an example of the same patient illustrating the differences between RTOG and 3Dose target volumes. Additionally, the glottic and supraglottic larynx (GSL) and inferior pharyngeal constrictor (IPC) were contoured as per Eisbruch et al [2]. These structures were not entered into the optimization algorithm but were reported.

**Table 1:** Patient Characteristics.

Patient	Primary Site	AJCC Stage
1	Hypopharynx	T2N2c
2	Supraglottic Larynx	T3N2c
3	Supraglottic Larynx	T3N0
4	Base of Tongue	T2N2a
5	Tonsil	T3N2b
6	Tonsil	T2N2b
7	Tonsil	T4N2c
8	Tonsil	T1N2a
9	Base of Tongue	T4N2c
10	Hypopharynx	T1N2b

Abbreviation: AJCC Stage = American Joint Committee on Cancer Staging 6^th ^Edition

**Table 2:** Contour Definitions.

	RTOG	3Dose
PTV-HD	(GTV + 1 cm) + 0.3 - 0.5 cm	GTV + 0.5 cm
PTV-ID	None	(GTV + 1 cm) + 0.3 - 0.5 cm
PTV-ED	((GTV + 2 cm) + elective nodes)) + 0.3 - 0.5 cm	Elective nodes + 0.3 - 0.5 cm

Abbreviations: RTOG = two dose level plan; 3Dose = three dose level plan; PTV-HD = high dose PTV; PTV-ID = intermediate dose PTV; PTV-ED = elective dose PTV; GTV = gross tumor volume

Plans were generated using the Eclipse treatment planning system (Eclipse 8.2.2, Varian Medical Systems, Palo Alto, CA). Seven equally spaced coplanar 6 MV beams with dynamic multileaf collimation were used for IMRT planning in nine patients; one patient was planned using nine equally spaced beams. The same optimization objectives for normal tissues used for the original RTOG plans were used for the 3Dose plans. PTV optimization objectives were initially identical between plans, but seven 3Dose plans needed revised objectives to meet an author-imposed coverage requirement of at least 95% of the volume of PTV-elective dose (PTV-ED) and PTV-intermediate dose (PTV-ID) receiving 100% of the prescribed dose. Target and normal tissue goals from RTOG 0522 are listed in Table 3. As per protocol, the PTV-HD received 70 Gy (2 Gy per fraction), and PTV-ED received 56 Gy (1.6 Gy per fraction). PTV-ID was prescribed 63 Gy (1.8 Gy per fraction). All plans were normalized such that 95% of the volume of PTV-HD received 70 Gy. The distributions of doses for different planning techniques were assessed for statistically significant differences using the paired t-test.

**Table 3:** Dose Constraints for Planning.

Structure	Maximum	Minimum
PTV-HD	<20% volume >110% Rx	<1% volume <93% Rx >95% volume >100% Rx
PTV-ID		<1% volume <93% Rx >95% volume >100% Rx
PTV-ED		<1% volume <93% Rx >95% volume >100% Rx
Parotid	Mean dose <26 Gy or 50% volume <30 Gy	
Cord	<0.03 cc >48 Gy	

Abbreviations: PTV-HD = high dose PTV; PTV-ID = intermediate dose PTV; PTV-ED = elective dose PTV; Rx = prescription dose

## Results

### Volumes

PTV-HD was significantly larger using the RTOG definition (p < 0.001) compared with the 3Dose definition, with a mean volume of 293.6 cc vs. 111.6 cc, respectively (Table 4). PTV-ED and the total target volume (i.e. PTV-HD + PTV-ID + PTV-ED) were also significantly larger using the RTOG definition (p < 0.001 for both comparisons), with a mean volume of 704.8 cc and 998.4 cc for the RTOG and 545.2 cc and 837.3 cc for the 3Dose definitions, respectively (Table 4).

**Table 4:** Planned Target Volume (PTV) Data.

	RTOG	3Dose	p
**Volumes (cc)**			
PTV-HD	293.6	111.6	<0.001
PTV-ED	704.8	545.2	<0.001
PTV Total	998.4	837.3	<0.001
**Doses**			
PTV-ED heterogeneity (%)			
110%	36.549	14.870	0.002
115%	15.086	2.716	<0.001
93% Target Coverage (%)			
PTV-HD	99.823	99.994	0.049
PTV-ED	99.449	99.705	0.06
Mean Target Doses (cGy)			
PTV-HD	7357.63	7240.90	0.01
PTV-ED	6099.57	5935.40	0.001
PTV Total	6468.31	6281.12	<0.001
Mean MUs	1675	1556	0.01

*Abbreviations*: RTOG = two dose level plan; 3Dose = three dose level plan; PTV-HD = high dose PTV; PTV-ED = elective dose PTV; PTV Total = sum of PTV-HD, PTV-ED, and PTV-ID; MUs = monitor units

### Doses

Coverage of PTV-HD was equivalent between the RTOG and 3Dose target definitions at the 100% prescription isodose (p = 0.86). Similarly, the volume of PTV-ED receiving 100% of the prescription was similar between definitions (p = 0.84). High dose heterogeneity at the 110% and 115% levels for the PTV-HD were similar between definitions, though there was a trend toward a smaller percentage of the PTV-HD receiving 110% of the dose for the 3Dose plans (p = 0.07). However, the heterogeneity at the 110% (p = 0.002) and 115% (p <0.001) levels for PTV-ED were significantly lower using the 3Dose definitions. The volume of PTV-HD receiving 93% of the prescription was significantly higher in the 3Dose plans (p = 0.049) (Table 4). The volume of PTV-ED receiving 93% of the prescription was higher in the 3Dose plans (p = 0.06). Mean doses to the PTV-HD and PTV-ED were both significantly lower in the 3Dose plans (Table 4). Additionally, the mean dose to the total target volume was significantly less in the 3Dose plans, 62.8 Gy compared with 64.7 Gy for the RTOG (p < 0.001). As a corollary, the mean monitor units necessary to deliver the plans was also less for the 3Dose definition plans, 1556 compared with 1675 for the RTOG (p = 0.01).

### Normal Structures

In the 3Dose plans, the mean dose to the parotid (p = 0.01) and mandible (p = 0.001) were less than in the RTOG plans (Table 5). Additionally, the volume of parotid receiving 30 Gy (p = 0.03) and the volume of mandible receiving 70 Gy (p = 0.03) were less in the 3Dose plans. Although doses to the GSL and the IPC were not specifically constrained, the mean doses in the 3Dose plans were still significantly less than RTOG plans for both comparisons (p < 0.001 and p = 0.001, respectively). When excluding patients with laryngeal or hypopharyngeal primary sites, the reductions in mean dose to the GSL and the IPC were quite large, 4.5 Gy and 5.3 Gy, respectively, in plans using 3Dose target volumes. Both the mean and the maximum dose to the brainstem were also less in the 3Dose plans (p = 0.001 and p = 0.01). However, there was no difference in the maximum dose to the spinal cord or the volume of tissue not otherwise specified receiving 70 Gy between the RTOG and 3Dose plans (Table 5).

**Table 5:** Normal Structure Doses.

	RTOG	3Dose	P
Parotid			
Mean (cGy)	2844.64	2631.30	0.01
V_30 _(%)	33.37	29.79	0.001
Mandible			
Mean	4430.08	4125.83	0.001
V_70_	6.779	1.772	0.03
GSL Mean	6604.05	6223.56	<0.001
IPC Mean	6136.40	5645.61	0.001
Brainstem			
Mean	1390.43	1300.14	0.001
Max	4609.93	4398.13	0.01
Cord Max	4284.28	4272.41	0.75
Tissue NOS V_70_	0.12145	0.04679	0.2

*Abbreviations*: RTOG= two dose level plan; 3Dose= three dose level plan; V_30_= volume receiving 30 Gy or more; V_70_= volume receiving 70 Gy or more; GSL= glottic and supraglottic larynx; IPC= inferior pharyngeal constrictor; Tissue NOS= tissue within body not contoured as target volume or organ at risk

## Discussion

Previous work from our group has suggested that early assumptions that called for large volumetric expansions on the high dose GTV may not improve locoregional control of tumors. This retrospective study found that volumetric expansions of ≤ 1.5 cm resulted in similar locoregional control as greater expansions [1]. In the report, direct GTV to PTV-HD expansions of 4-6 mm, without the use of a high dose clinical target volume, resulted in similar locoregional control as 10 - 15 mm expansions or > 15 mm expansions. A recent abstract from Sultanem et al., also reports satisfactory locoregional control and patterns of failure analysis without the use of a high dose clinical target volume [3]. Different institutional IMRT protocols may have an important impact on these findings regarding the high dose GTV-PTV expansion. For instance, the use of an intermediate dose PTV is an important factor to consider.

In this study we examined the possible dosimetric benefits of smaller target volumes. Utilizing a three dose level plan resulted in a more homogenous dose to the PTV-HD and PTV-ED, evidenced by improved 93% coverage of each volume with concomitant decreases in mean target doses and target volumes receiving 110% of prescription doses. This may be due to the smaller total target volume or the effect of the PTV-ID limiting dose to the periphery of PTV-HD and central portion of PTV-ED.

PTV-ID as used in the 3Dose plans serves a more important role than just improving heterogeneity, however. As shown in Table 2 the intermediate volume was identical to PTV-HD in RTOG plans. Any subclinical disease beyond the GTV is, by definition, microscopic. By replacing a high dose CTV with an intermediate dose CTV that receives a "microscopic dose" of 63 Gy, marginal failures may be avoided while providing improved toxicity outcomes. Additionally, Kashibatla et al. suggested that the use of concurrent chemotherapy provides a biological equivalent dose equal to a 12 Gy dose escalation in 2 Gy daily fractions [4]. Therefore, the delivery of 63 Gy with concurrent chemotherapy may result in comparable tumor control probability. For example, data from EORTC 24954 in advanced larynx and hypopharynx cancers would further substantiate this dose as 60 Gy split course radiotherapy with alternating chemotherapy provided equivalent outcomes to induction chemotherapy followed by 70 Gy of conventional radiotherapy [5]. Future investigations will be required to assess clinical outcomes with these proposed volumes and doses.

Blanco et al. and Chao et al. estimated that 4 - 5% of salivary function was lost for each additional 1 Gy in mean dose to the parotid [6,7]. Thus the finding that mean dose to the contralateral parotid was reduced 2.1 Gy in the 3Dose plans, from 28.4 Gy to 26.3 Gy (Table 5), may be a clinically significant amount. Four patients achieved a mean dose less than 26 Gy with RTOG plans, while the same 4 and one additional patient were under this threshold in the 3Dose plans. Additionally, the volume of the contralateral parotid receiving 30 Gy was reduced from 33.4% to 29.8%, though all patients met the < 50% parameter specified by RTOG 0522.

Currently, there is little data available regarding dose-volume parameters for the mandible in order to avoid osteoradionecrosis (ORN). Both Ben-David et al. and Studer et al. found very low rates of ORN (<1%) in a population treated with prophylactic dental care and IMRT [8,9]. However, Eisbruch et al. reported a risk of 6% in early stage oropharyngeal patients treated with IMRT on RTOG 0022 [10]. In two patients for whom dosimetry was available, ORN occurred in the sites of maximal dose, which approached 70 Gy in 30 fractions. It is therefore reasonable to assume that reducing the total dose and dose per fraction to the mandible will result in reduced rates of osteoradionecrosis. Using smaller target volumes, it is possible to significantly reduce the mean mandible dose (absolute decrease of 3 Gy) as well as higher doses to the mandible (absolute V_70 _decrease of 5%, Table 5).

Doses to both the GSL and IPC have been associated with late swallowing toxicity [2,11-15]. Mean doses to the GSL and IPC were reduced in the 3Dose plans, although these structures were not constrained in our planning process for either the RTOG or 3Dose plans. Further reductions should theoretically be possible in appropriate patients. For example, in the 6 patients without a primary site in the larynx or hypopharynx, the mean dose to the GSL was reduced 4.5 Gy, and the mean dose to the IPC was reduced 5.3 Gy using 3Dose target definitions.

Reductions in dose to the brainstem may reduce the incidence of nausea and vomiting during treatment [16]. In the current study, the mean dose to the brainstem was significantly less, though only by 90 cGy. The maximum dose to the brainstem was also reduced approximately by a mean of 2 Gy. These reductions likely are not clinically significant, though an improvement according to the "as low as reasonably achievable" principle.

## Conclusion

Investigations have suggested that patients may achieve equivalent locoregional control with wide target volumes as stipulated in various protocol regimens or more restrictive target volumes with additional dose levels as outlined herein. The present communication suggests that target volume reductions (with an additional intermediate dose level) may provide an enhanced therapeutic ratio as critical structures may be spared and patients may avoid debilitating toxicities. The refinement of IMRT treatments, with or without chemotherapy, for locoregionally head and neck cancer will require consideration of the ideal target volumes that allow tumor control while minimizing toxicity.

## Competing interests

James A. Bonner, M.D.: Occasional consultant/honoraria for Bristol-Myers Squibb Company, ImClone Systems, Inc., Eli Lilly and Company, Oncolytics, Sanofi-Aventis, and AstraZeneca. Speaker's bureau - Bristol-Myers Squibb Company.

There are no other competing interests.

## Authors' contributions

All authors participated in conceptualizing the project and writing the manuscript. MTM, PES and JJC captured and organized the data for the manuscript. All authors read and approved the final manuscript.
